# *‘You don’t throw these things out:’* an exploration of medicines retention and disposal practices in Australian homes

**DOI:** 10.1186/s12889-018-5753-6

**Published:** 2018-08-17

**Authors:** Fiona Kelly, Sara McMillan, Jean Spinks, Emilie Bettington, Amanda J. Wheeler

**Affiliations:** 1 Quality Use of Medicines (QUM) Network, School of Pharmacy and Pharmacology, Griffith University, Brisbane, Australia; 2 School of Pharmacy and Pharmacology, Menzies Health Institute, Griffith University, Brisbane, Australia; 3 Centre for Applied Health Economics, School of Medicine, and Menzies Health Institute Queensland, Griffith University, Brisbane, Australia; 4 Menzies Health Institute Queensland, Griffith University, Brisbane, Australia; 5 Faculty of Medical and Health Sciences, University of Auckland, Auckland, New Zealand

**Keywords:** Unwanted medicines, Medication safety, Medicine disposal, Quality use of medicines, Pharmacist

## Abstract

**Background:**

Consumers most commonly discard unwanted medicines in household rubbish or drains, however, there are global concerns over the extent, environmental impact and health risks. When consumers procure or store medicines for future use, this can impact negatively on quality use of medicines and consumer safety. We sought greater insight into the extent of these practices by exploring the volume and types of medicines in Australian homes, and self-reported practices related to medicine accumulation, use and disposal. This qualitative study formed part of a larger project that included a general population survey on household medicine disposal practices.

**Methods:**

Semi-structured telephone interviews were undertaken with a subset of respondents (*n* = 166) from the survey. Participants were eligible if they were experienced medicine users, i.e. used five or more prescribed, over the counter, and/or complementary and alternative medicines. Participants were asked to collect and name all medicines in their household; further detail was obtained about medicines used only when required or no longer used, such as expiry dates and quantity remaining. The quantitative data on the number and type of medicines stored at home were analysed descriptively. All interviews were transcribed verbatim and thematically analysed.

**Results:**

A total of 2301 medicines were identified as 1424 medicines not in everyday use (unused, unwanted, expired or when required) in 166 households, and 877 regularly used medicines by 119 participants. Medicines were often stored in multiple locations, particularly kitchens. Although accidental ingestion in children and pets and decreased efficacy were recognised health risks, this did not always translate to appropriate storage, usage or disposal practices. Individual risk-benefit assessments were applied to decisions to retain, use or dispose of medicines, including expired medicines.

**Conclusions:**

Inappropriate medicine storage, use, and/or disposal practices raises public health concerns, particularly as there is a free returned medicines scheme available, and that this particular participant group were considered experienced medicine users. Healthcare professionals must act to address consumer misconceptions around the quality use of medicines, including medicine retention, storage and disposal. Future research is warranted to explore consumer practices in this context and confirm these findings in a younger, or healthier population.

**Electronic supplementary material:**

The online version of this article (10.1186/s12889-018-5753-6) contains supplementary material, which is available to authorized users.

## Background

Medicine usage rates are increasing worldwide [1] and while timely access to medicines is important, there are global concerns over the extent of medicine waste from unused or unwanted medicines [2]. Concerns include public health and environmental impacts from inappropriate medicine disposal, for example inappropriate self-medication; accidental consumption by children; accumulation of active pharmaceutical ingredients in waterways as environmental pollutants; risk of antibacterial resistance and accidental poisoning of wildlife [3–8]. Strategies recommended to address these issues include consumer (general public) education about medicine costs and appropriate disposal practices [4]. Alternatively, the redistribution of unwanted medicines has been proposed as another option to reduce medicine waste [9, 10], a practice not yet sanctioned in countries such as the United Kingdom (UK) [11] and Australia [12].

Internationally, there is significant variability in how countries manage medicines disposal [13, 14], particularly between developing and developed countries. Across Europe there is variation in returned medicines schemes and in how the public are informed about safe medicines disposal [15]. Pharmacies are legally required to participate in selected countries, e.g. Denmark, France; and it is voluntary in others, e.g. Austria, Italy [15]. Review of Canadian and American schemes reveals significant variability in timing, e.g. 24 h availability, annual collection events; disposal points, e.g. pharmacies, secure drop-boxes; and funding sources, e.g. pharmaceutical companies, provincial government [14]. Medicines disposal in Afghanistan has been described as ‘substantially dysfunctional’ with a lack of resources and procedures [16]. Pharmaceutical wholesalers and manufacturers have allegedly avoided their obligations towards managing medicine waste in Serbia [17]. There are well-established, fully resourced return of unwanted medicine programs in Sweden [18], the UK [19] and Australia [20], all of which involve community pharmacies. In Australia, returned medicines are deposited in Return Unwanted Medicine (RUM) bins situated within pharmacies, which are then collected by pharmaceutical wholesalers and transported to registered incineration sites. The RUM Project is a national scheme established in 1998 and funded by the Australian Health Department as a quality use of medicines initiative. Between July 2000 and May 2018, Australian pharmacists have collected 8683.1 t of unwanted medicines, increasing from 19.6 to 66.4 t per month. However, despite the availability of this free national scheme to return medicines, the most common form of medicine disposal reported by Australian consumers is the rubbish bin followed by the toilet or drain [21, 22].

Consumer-led decisions are significant contributors to medicine waste, such as medicine non-compliance [23], obtaining medicines ‘just-in-case’ they are needed for future use [24], and self-initiated changes to prescribed treatment regimens [25]. Consumer actions towards medicines use and storage are also influenced by socio-demographic and contextual factors, such as cultural beliefs and the availability and costs of medicines. For example, in Ethiopia, limited quantities of conventional medicine were found in participant homes, which did not account for the use of traditional medicines [26]. Ekedahl et al. acknowledged that hoarding or over-supply was a significant contributor towards medicine wastage; this was within the context of a system allowing Swedish consumers to obtain more than three months’ supply of prescription medicines at a time [27]. Indeed, the action/s of health professionals in prescribing and supplying medicines should be recognised as contributors to the rates of unwanted or unused medicines. Doctors and pharmacists have a significant role to inform the public on safe medicines disposal practices [14, 22], yet the need to raise both public and health professional awareness of disposal schemes such as The RUM Project has been identified [28].

Given the plethora of studies that have explored the types of medicines returned to disposal collection points and why [29–31], or consumer opinions about disposal practices [16, 18, 24, 32, 33], it could be suggested that there is limited new information to be gained from this topic. However, medicine wastage remains a considerable public health issue. Limited studies have identified or accounted for medicines currently stored in the home [17, 23, 34]; no data on the volume of unused or unwanted medicines, e.g. full or near-empty boxes, has been sought and there is limited in-depth information into consumer self-reported practices. While concerns about poor medicine management practices have been identified for consumers with a range of chronic health conditions living at home in Uganda [35], overall, there has been limited focus on consumers using multiple medicines, a population that could be considered at greater risk of medicine mismanagement. In this study, we sought to explore the volume and type of medicines in Australian homes, as well as self-reported practices related to medicine accumulation, storage, use and disposal for consumers who could be considered experienced medicine users.

A two-stage mixed methods study into public awareness of The RUM Project and its effectiveness in reducing inappropriate medicine disposal was funded by the Australian Department of Health in 2016 [36]. Stage One involved a national audit of a representative sample of 423 RUM pharmacy collection bins to describe the nature of the medicines returned to community pharmacies [36]. Most of the disposed items were considered appropriate and not high-cost medicines [37]. In Stage Two, a representative general population online survey of 4302 people was conducted to explore whether they had medicines not in everyday use in their home and why, disposal practices and awareness of The RUM Project [36]. Participants who were not aware of the scheme were provided with some information and asked about their intended future use of the scheme [36]. Survey results established that there was limited awareness and inappropriate disposal practices [22]. This paper presents findings from a sub-section of Stage Two; interviews were conducted with a sample of survey participants who were using five or more medicines.

## Methods

### Study design

A purposeful and convenient sub-section of a representative survey sample of the Australian population by age, gender and geographical location were recruited by an external research panel company [22, 38]. Stage Two survey participants were eligible for interview if they were adults aged over 18 and used five or more medicines including prescribed, over the counter medicines and/or complementary and alternative medicines (CAMs) such as vitamins. Participants needed to speak English without the use of an interpreter. We estimated that 5% (*n* = 215) of the survey sample (*n* = 4302) should obtain an in-depth understanding of the medicine management and storage practices of consumers using multiple medicines, across a range of Australian homes. In the end, interviews ceased when data saturation was reached (*n* = 166; 3.9%), i.e. when no new information was offered. For the purpose of this study, medicines ‘not in everyday use’ is used to describe those medicines that are unused, unwanted, or expired. Medicines used on a when-required basis, e.g. over-the-counter analgesics, were also included in this case as there is a risk of accumulation of such medicines. A semi-structured interview guide (Table 1) was chosen to obtain specific medicine data, such as names, quantities and expiry dates of medicines not in everyday use, as well as participant views on medicine accumulation, storage, use, disposal and potential risks. The interview guide was informed by research on returned medicines [39, 40], the Stage Two survey [22] and feedback from the Project Advisory Panel. The interview guide was piloted with six members of the general population who were known to the research team personally, and who were using five or more medicines; minor amendments were made. University ethics approval was obtained (2016/449/GUHREC). Privacy concerns related to recording medicine details [4] were minimised as the research team did not obtain full participant details such as their residential address or prescriber name/s; all interviews were de-identified upon transcription.

**Table 1:** Key interview guide topics

Medicines in the home	Brand and/or generic name and storage location of medicines regularly used, medicines used as required and medicines no longer used.
Medicines not in everyday use	Formulation, quantity remaining and expiry date; disposal method/s; risks of keeping in the home.
Medicine supply	Frequency of supply, e.g. on-time, in-advance, just-in-case; household member/s who used the medicine; where the medicine was obtained, e.g. pharmacy, supermarket.
Medicine disposal	Self-reported disposal practices, views on and/or concerns related to what happens to medicines returned to a central location, e.g. a pharmacy.
Willingness to pay for safe disposal	Yes/No; explanation.
Demographic information	Gender, age, living arrangements, location, education, employment status, language spoken at home.

### Procedure

Eligible participants were invited to express interest in an interview at the completion of the Stage Two survey; their contact details were provided directly to the research panel company. The company provided a list of prospective interviewees to the research team separately to survey data to maintain confidentiality. Research team members phoned participants to schedule interviews when they were at home with access to their medicines. Participants were excluded when contact details were incorrect, they could not be contacted or they declined to participate.

To maximise opportunities for a full range of participant engagement, interviews occurred between 8 am and 8 pm on weekdays and weekends from September to October 2016. A research incentive was provided to participants by the research panel company, as per the terms of their engagement. Telephone interviews were conducted by three researchers at one of two University sites in Queensland, audio-recorded and independently transcribed verbatim. Prior to each interview the researcher obtained verbal consent and permission to audio-record the conversation. Participants were asked to physically locate the medicines in their home and had either gathered their medicines near the telephone or physically moved between storage location(s) in the home during the interview. Interviews were designed to be 15 min in duration and averaged 19.5 min (range: 8–81 min). Twice-daily debriefs to the entire research team were provided to facilitate discussions around data collection. Quality checking of a random sample of transcribed interviews, and all data entries of medicine information, was undertaken for reliability purposes.

### Data analysis

Data saturation was established after 166 interviews were completed and no new information was being offered. NVivo 11© software was used to assist with data management. Analysis of qualitative (open-ended) data was exploratory and data were thematically analysed by two researchers (FK, SM). Transcripts were read to assist with data familiarisation and frequent discussions were held between the two researchers due to the potential for bias; both were registered pharmacists. Units of data were coded into themes and data were compared and contrasted between transcripts. The interview guide topics were used as over-arching themes; sub-themes for each of these were identified in the data. Quotes have been coded by individual participant numbers throughout the text as P# and an ellipsis has been inserted to indicate omission of repetitive or unrelated text. Quantitative data collected on the medicines stored at home were entered into a purpose-built Microsoft Access® database for descriptive analysis. Medicines were classified by medicine schedule, e.g. prescription medicines and over the counter medicines, including those only available in pharmacies (Pharmacy and Pharmacist Only Medicines) and general sale or unscheduled medicines, including CAMs [41]. The therapeutic category for each medicine was identified using the classification system within the Australian Medicines Handbook [42].

## Results

The findings presented below describe the quantity and nature of medicines not in everyday use, storage locations and the proportion of expired medicines found in participants’ homes at the time of the interview.

Of the 3062 Stage Two survey participants who responded to the question about using five or more medicines, 40.8% (*n* = 1248) self-identified as high medicine users. Nearly half of these participants expressed an interest in a telephone interview (*n* = 608/1248; 48.7%), of which 166 went on to complete the interview (27.3%). This represented 3.9% of the Stage 2 survey population. Table 2 outlines the characteristics of interview participants and provides comparisons with the Stage Two general population survey sample [22]. Interview participants reflected an older sub-population of survey participants with 61.4% (*n* = 102/166) aged 55 years or older, and a greater proportion were retired. About two-thirds of interview participants lived in urban areas and the majority (80.7%; *n* = 134/166) resided in households of between two and five people (mean = 2), primarily with other family members (74.7%; 124/166). A total number of 2301 medicines were identified, including 877 medicines used on a regular basis for 119 participants; on average, seven medicines were used regularly, with one participant reporting a maximum of 36 medicines. The kitchen was commonly used to store medicines, followed by the bedroom and bathroom. Examples of storage spaces included, in or above the refrigerator, above the stove, in high cupboards, or on dining tables. Multiple locations were frequently mentioned, guided by storage requirements, e.g. refrigeration items; segregation by household member; differentiation between regular and ‘when required’ medicines, and as strategies to assist with medicine adherence. A minority of participants stated specifically that they used measures to restrict medicine access by children or pets.

**Table 2:** Participant characteristics

	Interview sample (2016) n (%)	General population Survey sample (2016) n (%)
TOTAL (N)	166	4, 302
Female	85 (51.2)	2203 (51.2)
Male	81 (48.8)	2099 (48.8)
Age range (years)^a^		
18–24	5 (3.0)	399 (9.3)
25–34	8 (4.8)	848 (19.7)
35–44	14 (8.4	826 (19.2)
45–54	33 (19.9)	785 (18.2)
55–64	45 (27.1)	660 (15.3)
65–99	57 (34.4)	784 (18.2)
State or territory^a^		
Australian Capital Territory	4 (2.4)	89 (2.1)
New South Wales	49 (29.5)	1383 (32.1)
Northern Territory	2 (1.2)	51 (1.2)
Queensland	38 (22.9)	868 (20.2)
South Australia	10 (6.0)	306 (7.1)
Tasmania	3 (1.8)	91 (2.1)
Victoria	42 (25.3)	1081 (25.1)
Western Australia	15 (9.0)	433 (10.1)
English spoken at home^ab^	158 (95.2)	4026 (93.6)
Educational experience^a^		
Year 9,10 or below	23 (13.9)	522 (12.1)
Year 11 or 12	31 (18.7)	823 (19.1)
Tertiary study^c^	106 (63.8)	2957 (68.8)
Employment status		
Retired or pensioner	64 (38.6)	980 (22.8)
Working part- or full-time	51 (30.8)	2371 (55.1)
Unemployed	10 (6.0)	348 (8.1)
Student	3 (1.8)	187 (4.3)
Self-employed	9 (5.4)	236 (5.5)
Other^d^	23 (13.9)	180 (4.2)

^a^Missing data – not all respondents provided this information

^b^Other language responses included: Chinese, Filipino and Italian

^c^Tertiary study includes technical college and university

^d^Other Employment status responses included: homemaker, n = 5; disabled/on disability pension, n = 11; voluntary work, n = 2; and on workers compensation, n = 1

Participant self-reported accumulation of medicines not in everyday use, obtaining medicines ‘just in case,’ views on the risks of keeping unwanted medicines, use of expired medicines, and medicine disposal practices are described in the following sections. Additional quotes are provided in Additional file 1: Table S1.

### Medicines not in everyday use stored at home

At the time of the interview, a total of 1424 medicines not in everyday use were stored at home. Although 54.8% of participants (*n* = 91/166) initially stated that they were not currently storing any unwanted medicines, many discovered unused or expired medicines during the interview. Some participants articulated concerns over the quantity of medicines stored, others used the interview as an opportunity to clean out their accumulated medicines:

*“I’ve got Betnovate*® [betamethasone valerate 0.1%] *cream and that’s expired March 2015. This is cleaning out my cupboard nicely…Got a cream called Elocon*® [mometasone furoate 0.1%]*. Just looking at the expiry date on that one… 2012. See here I thought I was so good. I’d thrown all my other stuff out and I’m finding all these ones.”* (P 548)

About two-thirds of the medicines not in everyday use (62.7%, 893/1424) were available over the counter without a prescription; this included 266/413 (64.4%) expired medicines within this category (Table 3).

**Table 3:** Unwanted or ‘when required’ medicines reported in households

	Total medicines by category n (%)	% Expired medicines by category n (%)
TOTAL (N)	1424^a^	413^c^
Controlled medicines^b^	31 (2.2)	7 (1.7)
Prescription only medicines^b^	418 (29.4)	113 (27.3)
Pharmacist only medicines^b^	117 (8.2)	30 (7.3)
Pharmacy medicines^b^	274 (19.2)	72 (17.4)
General sale medicines^b^	374 (26.3)	121 (29.3)
CAMs	128 (8.9)	43 (10.4)

^a^Includes 82 items that were classified as: international medicines (n = 15) or unknown (n = 67)

^b^Controlled medicines require the greatest level of restrictions in related to storage in a locked safe, supply and documentation (e.g. morphine). Over the counter medicines include Pharmacist Only Medicines, Pharmacy Medicines and general sale medicines. Pharmacist Only Medicines are restricted to pharmacy only sale and require the involvement of pharmacists in the sale. Pharmacy Medicines are restricted to pharmacy only sale whereas general sale medicines are available from supermarkets and other retail outlets

^c^Includes 27 items that were classified as: international medicines (n = 6) or unknown (n = 21)

*CAMs* complementary and alternative medicines

The most common therapeutic medicine category was analgesics (23.7%, 337/1424), followed by gastrointestinal-related medicines (11.7%, 166/1424), then respiratory (10.0%, 142/1424) and dermatology (9.5%, 135/1424). Over the counter medicines represented 60.7 to 81.7% of the medicines within each category. About a third of the gastrointestinal- (*n* = 50/166) and dermatology-related medicines (*n* = 45/135) were prescription medicines; more than double that of the analgesics and respiratory categories. The majority of medicines not in everyday use (85.2%; *n* = 1213/1424) were opened packets, with 70.4% (*n* = 1002/1424) intended for use by the participant and a third (*n* = 472) by family members. Reasons why stored medicines were no longer used included prescriber changes and/or autonomous decisions to cease use due to lack of effect, unwanted side effects or improved symptoms through lifestyle management:

*“He* [doctor] *increased the dosage* [antipsychotic]. *I had new prescriptions with increased amounts. So these just got left in my bedside table.”* (P 542)

*“Yes I ceased it* [gout medication] *because I didn’t think it was making any difference to my life; I was sick of taking tablets.”* (P 457)

Stored ‘when required’ medicines included those used for minor ailments, treatment for symptom flare-up of episodic illnesses, prescribed medicines kept ‘just in case’ after they were no longer needed, e.g. antibiotics; or medicines used by multiple household members:

*“…Well, there are three packets* [paracetamol] *there because I buy this in bulk…this is an item that everyone uses in the house and I buy three or four boxes at a time*…” (P 711)

### Medicine accumulation ‘just in case’

When researchers asked whether participants ever deliberately obtained prescribed medicines ‘just in case,’ 90% (*n* = 117/130) of participants reported that they did not, yet they reported that they did store medicines for future use; for either similar or alternative purposes to that originally prescribed. Factors that influenced accumulation included living or working away from home; ensuring adequate supply always on hand; impending travel and episodic illnesses such as migraines:

*“I think when we've gone overseas; we've had some stuff that was just in case, like Gastro-Stop®* [loperamide], *and things like that.”* (P 1659)

*“...The chemist basically issued a repeat and the first dose in one go because I wasn't too well at the time so it saves you coming back, there's your repeat, but I never used the repeat so that box* [amoxicillin] *is full. The expiry is 12/15…”* (P 711)

*“Mainly the Imigran®* [sumatriptan]*, I don’t like to run out of those, they treat migraines. I don’t like to run out of any of them*.” (P 169)

A small number of participants stated they were unlikely to discard medicines due to frequent prescribing changes, to limit wastage, or because they were forgotten, e.g. at the bottom of cupboards. When dose strengths were increased, some participants reported that the doctor advised them to double-dose to avoid wastage. A few participants viewed medicines as items that you do not discard:

*“…I've stopped using it* [Ovestin® Vaginal Cream]*, basically, but I've still got the tube because you don't throw these things out.”* (P 2280)

There were examples of obtaining or retaining antibiotics as an ‘insurance policy’ against future infections with no mention of potential for incorrect self-diagnosis or antimicrobial resistance:

*“…I'll take the first course completely, and then I might only take two-thirds of the rest of the second course and not complete it, and that means I might have a few spare. The way I see it, if I get something that looks pretty bad, like a bacterial infection in the throat or whatever, I don't have to go racing to the doctor in the middle of the night…”* (P1240)

“*…I haven't disposed of it* [Norfloxacin] *because I had acute prostate episodes and that was prescribed to me, that helps. I kept it just in case it happens again...it's kind of a security blanket...”* (P 1711)

Participants who did not retain antibiotics ‘just in case’ felt that they took enough regular medicines already, did not want to use antibiotics unwisely, or intended to purchase medicines overseas:

*“…I wouldn't take them* [antibiotics] *just in case, no they're no good taking them just in case.”* (P 302)

*“I generally don't take medicines* [overseas] *with me because I know like in Vietnam…we can get everything we need there, like antibiotics and antihistamines and so forth.”* (P 2252)

### Medicine expiry and perceived issues with storing medicines not in everyday use at home

The expiry dates provided for stored medicines spanned 30 years from 1991 to 2021: 37.0% (*n* = 413) had expired as of the 31st October 2016 and 12.8% (*n* = 143) expired within six months of that date. Higher proportions of expired, or almost expired, medicines (58.8%; *n* = 177/301) were reported for general sale medicines or CAMs than prescription or pharmacy medicines (44.7%; *n* = 317/709). Participants stated that expiry dates were sometimes difficult to read, were obscured by dispensing labels or were missing when primary packaging had been discarded.

Some participants were surprised to find expired medicines in their home and/or were unaware that medicines do expire. Some participants assumed medicines were in date if recently dispensed, or questioned if medicines expired:

*“I know I got it earlier this year so it ought to be all right. I've looked on the container and I just can't see any expiry date on it.”* (P 2182)

*“…I'm trying to look what's on it* [bisacodyl 5mg]*. Yeah, 2009. Do they go out of date, really?”* (P 2183)

Participants generally associated medicine expiry with reduced efficacy rather than safety concerns:

*“I guess primarily if it was really out of date it wouldn’t be as effective for the purpose it was intended for so it might create issues. Especially if you take something in an emergency and it doesn’t do what it’s supposed to.”* (P 945)

Examples of expired medicines being used included analgesics, pseudoephedrine (decongestant), chloramphenicol (antibiotic) eye preparations and topical corticosteroids. Accepted timeframes for use of expired medicines by participants varied from just expired, to a year or longer; there was greater use of expired topical medicines reported and expired prescription medicines were associated with higher risks. Overall, participants appeared to make individual risk-benefit calculations with respect to expired medicines:

*“With the Panadeine®* [paracetamol 500mg, codeine 8mg] *and the Sudafed®* [pseudoephedrine 30mg] *I would normally take them and not worry about the expiry date…The Clexane®* [enoxaparin sodium] *slightly different…I would go visit my doctor and make sure that the Clexane® was a valid in stock prescription and I would actually bring the out of date ones back to the chemist…”* (P 58)

When asked about possible issues associated with storing medicines not for everyday use at home, participants cited safety risks as the main concern, particularly unintentional poisoning in pets and children:

*“Look, it just depends on how careful people are. I mean, particularly if there are young children around then I would be concerned. You’ve got to keep the stuff secure, either out of height or sight of little people, I mean kids…”* (P 139A)

Other issues included confusion and medicine duplication if multiple generic brands were stored; self-diagnosis and self-medication without medical advice; and sharing of medicines:

*“The patient themselves can get confused, particularly when you’ve got all these generic medicine brands at eye level today and be taking two of one thing! So, it’s a bit of a hazard, I think, to have unused medicines and unwanted medicines in the home.”* (P 65)

*“People may be inclined to, later on down the track, self-medicate without proper advice.”* (P 1596)

*“If they're not properly stored in the house, children could easily pick them up and eat them. You might be more tempted to share. I've got this, I'm sure, they worked for me I'm sure they'll work for you. You might hand them out to other people, your friends, relatives, family, whatever.”* (P 2268)

A small number of participants highlighted the risk that medicines not for everyday use could be misused, re-sold or instigate break-ins.

### Medicine disposal

Many interview participants voiced an intention to return medicines to a pharmacy after finding out about The RUM Project at the end of the Stage Two general population survey:

*“ Well, up until a couple months ago I think I just tossed them in the bin, because I've never been told before that I actually have to hand them in at the pharmacy…”* (P 2895)

Even so, 36 interview participants described a combination of disposal practices, which appeared to be influenced by the formulation or schedule of the medicine. Participants discussed that they were more likely to return prescription tablets to the pharmacy and discard ointments, liquids, CAMs or general sale medicines, e.g. antacids, lozenges and cough mixtures, in the rubbish or down the drain:

*“If it's liquid, I throw it down the toilet. If they are other pills, I throw them in the garbage. I wrap them up in a plastic bag and throw them.”* (P 2001)

*“Prescription ones we take down to the chemist. The other ones we obviously hoist in the rubbish bin.”* (P 270)

Participants applied individual risk assessments to guide disposal (Additional file 1: Table S1), considering potential toxicity, perceived efficacy, inappropriate use, convenience and potential for diversion of medicines for manufacture of illicit drugs:

*“The medicines that if somebody could go into my bin and get hold of and take and make themselves sick or kill themselves on, I’ll take to the chemist…But if it’s something like cream that I’m sure nobody’s going to eat…I’d just throw it out.”* (P 598)

*“If I thought it was addictive or could be used by druggies or used to make other things that they're doing nowadays definitely take it to the pharmacy.”* (P 605)

Disposal of medicines in the household rubbish was reported more frequently by younger participants (18 to 44 years), yet a greater proportion of people aged 45 years or older discarded medicines down the drain. Return of medicines to the pharmacy was more prevalent amongst people living in rural locations, aged 45 years or older or those with recent healthcare work experience.

The majority of participants assumed there was a “*proper process”* (P 1381) that pharmacists applied to disposal of returned medicines, yet understanding of actual processes varied:

*“I guess I'm not particularly concerned if I return them* [medicines] *to a chemist. What they do with them, I'm sure they do whatever needs to be done, the right thing.”* (P 2168)

“[The pharmacy] *probably flush it down the toilet or give it back to the manufacturer. They might be able to make a claim on it…”* (P2465)

About a third of participants discussed recycling or resale of medicines to reduce wastage, provide access to medicines for people who cannot afford them, or to send to developing countries. However, there was ambivalence over the reuse of medicines, particularly expired medicines:

*“I think some medicines would be appropriate* [to send overseas]; *I don’t think they are useless completely when they’re past their use by date. Others maybe not, I’d rather they be disposed of.”* (P 945)

*“…My thinking is well, why do they get our cast-offs* [medicines]…*They probably deserve just as good as we have….We shouldn't just necessarily throw our rubbish at them. We should sort of fund proper supplies…but I suppose on the other hand it's better to have something rather than nothing...”* (P 2252)

One participant stated that if medicines were to be re-used, they might as well just keep them themselves.

## Discussion

A range of medicines not for everyday use are stored in multiple locations in Australian homes, and self-reported medicine storage, usage and disposal practices have direct implications for medicine quality, as well as environmental and health risks for individuals and other household members. Whilst participants acknowledged accidental ingestion and reduced efficacy as key health risks of storing and using such medicines, this did not always translate to appropriate storage in the home. The majority of the medicines not in everyday use were found to be those purchased without a prescription. Participants did not intentionally stockpile prescribed medicine, yet they did retain certain excess medicines ‘just in case’ of future need, even when these medicines had expired. Although findings align with other research on medicine storage and disposal practices [21, 22], we provide additional insights into participant rationales for ‘just in case’ medicine use, as well as expired medicines. Overall, the propensity of participants to use individual and varied risk-benefit strategies to guide these behaviours was prominent.

Multiple storage locations were used to support medicines adherence and provide easy access to commonly used medicines. There were concerns that storing different generic versions of prescription medicines in multiple locations could create confusion and lead to medicine duplication. These add to existing concerns over consumer confusion related to generic brands [43]. Additionally, if some of these storage locations are infrequently accessed, medicines are more likely to expire. However, the potential for decreased efficacy of medicines stored in bathrooms, and the risk of accidental ingestion by children when medicines are kept within easy access, such as dining room tables, was not always considered by participants. Although the majority of participants in this study were over the age of 55 and unlikely to have children living at home, as potential grandparents, they may be placing visiting grandchildren at risk with current medicine storage practices. While participants were aware that medicines not for everyday use could be hazardous to children, to what extent this translated to preventative action when grandchildren or others visited can only be speculated. While some research into the impact of grandparent actions on the physical wellbeing of grandchildren is available [44], a focus on medication safety in terms of medicines not for everyday use is lacking and therefore, necessary.

Participants demonstrated varying degrees of awareness about the volume of expired medicines in the home and the associated safety risks. Whilst keeping expired medicines is not new, insight into continued use is limited. We know that financial burden can influence collection of prescribed medicines [45], but greater understanding of whether it contributes to continued use of expired medicines is warranted. Expired prescription medicines have previously been removed from the homes of consumers considered to be at-risk of medicine misadventure, i.e. those over the age of 55 with chronic health conditions [6]. Healthcare professionals cannot presume that high medicine users, as seen in our study population, are knowledgeable in terms of the quality use of medicine or medicine safety. Participants’ views that medicines were safe and effective to use well beyond expiry, particularly topical medicines, are similar to that found by Dawood et al.; consumers believed that the expiry date of ointments, gels and syrups could be extended by keeping them in the refrigerator [32]. However, reduced effectiveness may have negative health implications if expired medicines with decreased efficacy are used to manage chronic health conditions. Our findings highlight the need for further health campaigns, regular review of household medicines and more routine reminders from health professionals than what currently occurs [22], specifically for consumers using multiple medicines. A few participants mentioned the risk of sharing medicines not for everyday use; this builds on existing evidence of medicine sharing [46, 47], but does not specifically explore sharing of expired medicines. Qualitative comparison of consumer views on, and decisions about sharing current medicines, those medicines not for everyday use, and expired medicines would provide additional insight.

Individual risk-benefit assessment appeared to guide accumulation of excess medicines for ‘just in case’ use. The key benefit was to have medicines on hand for ease of use, which is appropriate for episodic conditions such as migraines, yet inappropriate if retaining antibiotics to self-treat future infections, particularly viral infections. Inappropriate antibiotic use was reported in another survey of unused or expired medicines [48], and few people were concerned about resistance or treatment failure when sharing antibiotics with family or friends [46]. Microbial resistance is a significant public health concern and public health campaigns emphasise inappropriate antibiotic use in viral infections such as colds and influenza. However, consumers may not translate this public health message to other infections. Another finding from our study was that the majority of unwanted or expired medicines found in homes were over the counter medicines. It may be that consumers perceive over the counter medicines to be safe [49], or safer than prescription medicines [50], although we did not explore such perceptions. As over the counter medicines traditionally involve less input from health care professionals, e.g. if purchased in a supermarket, further emphasis on the message that ‘*OTC medicines should be treated with the same care as prescribed medicines* [51]’ is suggested.

Medicine disposal practices varied and 20% of participants reported a combination of both returning medicines to the pharmacy and disposing via household garbage or drains. Such practice was guided by individual perceptions of health and safety rather than the risk to the environment. Indeed, how consumers dispose of other perishable goods could also influence medicine disposal; liquids were more likely to be poured down the drain similar to other fluids, and tablets placed in the bin like other solids such as food scraps. On a positive note, increasing consumer awareness of pharmacy services for disposal of medicines not for everyday use can improve intended disposal practices [3]. This was identified in interviews, which perhaps reflects an educational effect of the Stage Two survey that could be extended to additional populations. Furthermore, there was significant trust in pharmacists to safely dispose of returned medicines tempered by ambivalence over how this process actually occurs and whether medicines would be reused overseas. Government funding of medicines disposal via The RUM Project supports appropriate disposal, unlike countries such as New Zealand where some pharmacists shoulder this financial burden [52]. Variability in disposal practices and views on the acceptability of medicine reuse further highlight different risk-benefit strategies and potential misconceptions. Health campaigns and grass-roots strategies, e.g. clean out your medicines day, reminder stickers, could encourage regular return of unwanted household medicines; increase awareness of The RUM Project (http://www.returnmed.com.au); address misconceptions and promote appropriate medicine storage, use and disposal of unwanted and expired medicines.

Study limitations include: interview data represents a snapshot in time and omits those medicines stored in other locations such as the workplace [5], or the potential for people to misread expiry dates [53]. We spoke to a single member of the household and there was potential under-reporting of medicines due to privacy concerns, and self-reported practices did not necessarily reflect those of other household members. We did not explore the influence of multiple prescribers or chronic illness morbidity on medicine management practices [35] and these limitations identify areas for more targeted exploration of medicine accumulation, use and disposal. Interviews were conducted with consumers using multiple medicines, the majority of which were over the age of 55 years. How these results differ from a generally healthy person, e.g. a person who use medicines sparingly, a person who uses less than five medicines a day, or a younger person, is unknown. In general, ‘when required’ medicines are not considered as unwanted medicines; however, this study identified that such medicines are also at risk of accumulation in households. A key strength of this study was the nationwide sample of people self-reporting medicines storage, usage and disposal practices to reveal the individual risk-benefit assessments that guide these behaviours. While other studies have used in-home visits to inventory consumer medicines [25, 35, 54, 55] these have been restricted to discrete, often local, populations, unlike our nationwide representative sample. Additionally, an interview protocol was developed to ensure consistency between interviewers and limit potential for bias introduced by their pharmacy background; this enabled researchers to identify important insights into this subject area.

## Conclusions

Australian consumers who could be considered experienced medicine users are storing medicines not for everyday use at home. The use of such medicines, including those which are expired, is not always appropriate and is guided by variable risk-benefit assessments and this has implications for individuals and households, the broader health system and the environment. Further research is needed to confirm these results with a younger, or healthier, population. Greater understanding of the underlying basis and significance of this is needed to identify and effectively address common misconceptions through consumer health campaigns or grass-roots strategies.

## Additional file

Additional file 1: **Table S1.** ‘Additional views on medicine storage and disposal practices’ (Table S1) has been included to provide readers with additional context on the views and self-reported practices of participants through quotes aligned to key themes identified in the data. (DOCX 41 kb)

## Abbreviations

CAMs: Complementary and Alternative Medicines
RUM: Return of Unwanted Medicines
UK: United Kingdom
