# Supplementary material for: ‘You don’t throw these things out:’ an exploration of medicines retention and disposal practices in Australian homes
Source: BMC Public Health. 2018 Aug 17;18:1026. doi: 10.1186/s12889-018-5753-6 (PMC6098630; doi:10.1186/s12889-018-5753-6)
Supplement: Supplementary file 1 — Table S1. ‘Additional views on medicine storage and disposal practices’ (Table S1) has been included to provide readers with additional context on the views and self-reported practices of participants through quotes aligned to key themes identified in the data. (DOCX 41 kb) [file 12889_2018_5753_MOESM1_ESM.docx]

**Table S1: Additional views on medicine storage and disposal practices**

| **Factors** | **Participant quotes** |
| --- | --- |
| **Loss of efficacy** | *“… I just don't see the point of keeping anything past the time. They're the best before for a reason…”* (P 1423) |
| **Potential for misuse or harm** | *“I’d rather they’re* [medicines] *all going there* [pharmacy] *and not being left in bins where other people can get their hands on and misuse them. Like children get hold of them or people take them to try and get high or whatever.”* (P 332)  *“Years ago I made a mistake of throwing some* [medication] *down the toilet, before we knew how it affected the waterways and that sort of thing. I’m going back 30 years. I found my son, my two-year-old, in there and I didn’t know whether he’d actually taken one of the tablets or not. So, I never, ever throw any down the toilet ever again.”* (P548) |
|  | *“The Panamax®* [paracetamol]*, yes* [would return to the pharmacy]*. The Strepsils®* [antiseptic throat lozenge] *I would probably just throw in my rubbish because I'm sure what's in them.”* (P 333) |
| **Being environmentally responsible** | *“I want to do it safely and I know other people tell me that they flush it down the sink, but I just say that just goes out into the ocean.”* (P 2788*)* |
| **Privacy and ethical concerns with returning medicines** | *“…I wouldn't think that ethically that they would reuse expired medicines but it is a bit of a concern to, yeah, to know exactly what does happen to them.”* (P 1940)  *“A while ago, I would’ve said I’d have privacy concerns. Or being judged on what sort of medications…you’re handing in. But these days I’m a lot more relaxed about it.”* (P 494) |
